# Accounting for Nulliparity in the Prediction of Hypoxic-Ischemic Encephalopathy Using Cardiotocography

**DOI:** 10.1109/bhi58575.2023.10313456

**Published:** 2023-11-14

**Authors:** Johann Vargas-Calixto, Yvonne W. Wu, Michael Kuzniewicz, Marie-Coralie Cornet, Heather Forquer, Lawrence Gerstley, Emily Hamilton, Philip A. Warrick, Robert E. Kearney

**Affiliations:** Department of Biomedical Engineering, McGill University, Montreal, QC H3A 2B4, Canada.; University of California, San Francisco, CA 94158, USA.; Kaiser Permanente, Oakland, CA 94612, USA.; University of California, San Francisco, CA 94158, USA.; Kaiser Permanente, Oakland, CA 94612, USA.; Kaiser Permanente, Oakland, CA 94612, USA.; McGill University, Montreal, QC H3A 2B4, Canada, and with PeriGen Inc., Montreal, QC H4Z1E8, Canada.; McGill University, Montreal, QC H3A 2B4, Canada, and with PeriGen Inc., Montreal, QC H4Z1E8, Canada.; Department of Biomedical Engineering, McGill University, Montreal, QC H3A 2B4, Canada.

## Abstract

Nulliparous pregnancies, those where the mother has not previously given birth, are associated with longer labors and hence expose the fetus to more contractions and other adverse intrapartum conditions such as chorioamnionitis. The objective of the present study was to test if accounting for nulliparity could improve the detection of fetuses at increased risk of developing hypoxic-ischemic encephalopathy (HIE). During labor, clinicians assess the fetal heart rate and uterine pressure signals to identify fetuses at risk of developing HIE. In this study, we performed random forest classification using fetal heart rate and uterine pressure features from 40,831 births, including 374 that developed HIE. We analyzed a two-path classification approach that analyzed separately the fetuses from nulliparous and multiparous mothers, and a one-path classification approach that included the clinical variable for nulliparity as a classification feature. We compared these two approaches to a one-path classifier that had no information about the parity of the mothers. We also compared our results to the rate of Caesarean deliveries in each group, which is used clinically to interrupt the progression towards HIE. All the classifiers detected more fetuses that developed HIE than the observed Caesarean rate, but accounting for nulliparity did not improve performance.

## I. Introduction

According to the World Health Organization, in 2019, birth asphyxia and intrapartum complications accounted for about 24% of neonatal deaths [1]. One of the birth asphyxia-related complications is hypoxic-ischemic encephalopathy (HIE). Neonates with moderate to severe HIE are at higher risk of catastrophic consequences such as permanent motor and cognitive disorders and even death [2]. The consequences of HIE are particularly severe in low- to middle-income countries where 31% of infants with HIE die in the neonatal period [3].

Uterine contractions are inherent to the process of labor and delivery. Although contractions can cause intermittent periods of fetal hypoxia, the fetus has several physiological compensatory mechanisms so that generally there are no harmful effects. However, if labor is prolonged, involves overly frequent and strong contractions, or there is an impaired fetal response, the fetus may not compensate adequately and can develop fetal acidosis and possibly subsequent HIE. Certain clinical risk factors can make some labors more stressful. For instance, labor in nulliparous mothers is longer and it is associated with a higher prevalence of infection which is a contributory factor for HIE [4].

Intrapartum cardiotocography (CTG), consisting of fetal heart rate (FHR) and uterine pressure (UP) signals, is used to monitor fetuses and detect those at risk of acidosis and HIE. If clinicians detect an infant with worrisome CTG, they will perform a Caesarean delivery to interrupt the progression towards HIE. Unfortunately, the visual interpretation of these signals has high intra- and inter-observer variability, that reduces their discriminability [5]. Previously, we showed that a random forest classifier could detect fetuses at increased risk of HIE from CTG and recommend clinical intervention [6].

In this study, we hypothesized that accounting for nulliparity could improve the interpretation of FHR and UP patterns. As nulliparous labors tend to be longer and more challenging for the fetus, it is reasonable to expect that knowing whether a pregnancy is nulliparous or multiparous should help a classifier learn better the particularities of each type of labor and improve the overall detection of HIE.

## II. Clinical Data

This retrospective study used data collected in 15 Kaiser Permanente Northern California hospitals between 2011 and 2019. The data includes CTG signals, and pregnancy and labor clinical variables from 246,968 singleton births with a gestational age of 35 weeks or older and no congenital defects. We focused this analysis on a subset of 40,831 births that had cord or neonatal blood gases available within the first 2 hours of age. We divided the data into three groups: (1) 37,546 healthy infants that had cord pH > 7 and cord or infant base deficit < 10 mmol/L; (2) 3,056 infants with fetal acidosis defined by cord pH ≤ 7 or cord or infant base deficit ≥ 10 mmol/L, and no evidence of encephalopathy; and (3) 374 infants that developed HIE defined by acidosis and clinical evidence of encephalopathy. Neonatal encephalopathy was defined by an abnormal neurologic exam between 1 – 6 hours of age that persisted beyond 6 hours of age, was accompanied by seizures, or treated with therapeutic hypothermia.

## III. Methods

We assessed the association between nulliparity and acidosis or HIE using the Chi-square test of independence and calculated the relative risk (RR) and 95% confidence interval (CI). Then, we processed the CTG signals to extract features and trained a random forest classifier.

### A. FHR and UP processing

FHR was sampled at 4 Hz while UP was sampled at 1 Hz and then up-sampled to 4 Hz. We used PeriCALM Patterns, a software system from PeriGen Inc., to preprocess the CTG [7, 8]. Gaps shorter than 15 s in the FHR were linearly interpolated. Then, the FHR was filtered with an ensemble of low-pass, high-pass and Karhunen-Loève filters [7, 8]. We included signals up to 12 hours before delivery. PeriCALM Patterns detected and labeled CTG events such as the UP contractions and the baselines, accelerations, and decelerations of the FHR. The CTG signals were divided into 20-minutelong epochs and those with less than 80% valid samples were excluded from analysis. Finally, for each epoch, we counted the transitions between FHR and UP events and determined the dwell time of the events, defined as the time spent in one event before transitioning into another [6].

### B. Epoch classification

We divided the data into training and testing sets: 90% of the individuals were used for training and 10% for testing. Then we trained four *C*_*all+np*_ on the epochs from all the fetuses in the training set and that included the clinical variable for nulliparity classifiers on FHR and UP features: (i) *C*_*all*_ on the epochs from all the fetuses in the training set, (ii) as a classification feature, (iii) *C*_*np*_ for fetuses from nulliparous mothers, and (iv) *C*_*mp*_ for fetuses from multiparous mothers.

The classification problem was defined as binary classification between the healthy group and a pathological class containing individuals from the acidosis and HIE groups. We used random forest classifiers consisting of 2,500 trees and under-sampled the majority class for each tree to achieve class balance; for every two healthy individuals, there was one from the acidosis and one from the HIE groups. We used the out-of-bag predictions from the trained model for validation and hyperparameter selection. The output of the epoch classifier was the posterior probability *p*_*PAT*_ that the epoch belonged to an infant from the pathological class. When *p*_*PAT*_ was larger than a threshold *t*_*PAT*_, the epoch was labeled as pathological, and an alert was issued for the infant. We used directly *C*_*al*_ and *C*_*all+np*_ on all the epochs in the test set. However, the classifiers *C*_*np*_ and *C*_*mp*_ required the algorithm to first examine nulliparity to determine which classifier to use for testing.

### C. Decision support system

Data from single individuals could have up to 36 epochs. We aggregated the predictions of multiple epochs to determine the final output. We aggregated the predictions of *C*_*all*_, *C*_*all+np*_, *C*_*np*_, and *C*_*mp*_ separately.

As in [6], for each individual the system examined a sliding window of 100 min, starting at the earliest epoch before delivery. If more than two epochs were missing from the 100 min window, the window was ignored, and no analysis was made until the algorithm found a window that had 3 or more valid epochs. The decision support system required that all the available epochs within the moving window had issued an alert to recommend clinical intervention. Using the out-of-bag predictions, we selected the optimal threshold ${\hat{t}}_{PAT}$ such that the false positive rate of the recommendations matched the Caesarean delivery rate in the healthy group. The Caesarean delivery rate varied according to the population used to train the classifier. Finally, we determined the rate of recommended intervention in the test set. The system could recommend interventions up to 40 minutes before delivery. Decisions made after that are too late to permit effective clinical interventions. Using the predictions of the *C*_*all*_, *C*_*all+np*_, *C*_*np*_ and *C*_*mp*_ separately, we optimml:mized the threshold ${\hat{t}}_{PAT}$ which led to the four decision support systems *DS*_*all*_, *DS*_*all+np*_, *DS*_*np*_, and *DS*_*mp*_ respectively.

### D. Assessing performance

We trained the classifiers and decision support systems on 𝑛 = 100 independent samples of the training and test set to generate distributions of the performance metrics. We used the median and its CI to compare the rate of intervention recommendation of *DS*_*all*_, *DS*_*all+np*_, *DS*_*np*_, and *DS*_*mp*_ on each of the study groups. The CI of the median was defined as

(1) $$\text{media}n_{CI}=\text{median}(x)\pm 1.57*\left(\frac{iqr(x)}{\sqrt{n}}\right),$$

where *x* was the intervention recommendation rate for each group, *n* was the number of samples, and *iqr* was the interquartile range of the rate of intervention recommendation in each group. We tested if the medians of recommendations were different using the Wilcoxon rank-sum test. Differences were deemed significant for p < 0.05.

## IV. Results

### A. Clinical factors

In our study group, 58.4% of births were from nulliparous mothers. Table I shows the distribution of individuals from each group between nulliparous and multiparous pregnancies. Nulliparity was significantly associated with an increased risk of developing HIE exclusively (RR = 1.43, CI = [1.16 – 1.78], p < 0.001), and an increased risk of developing acidosis (RR = 1.31, CI = [1.23 – 1.40], p < 0.0001).

### B. Decision support system

Table II shows the distribution of recommendations and the Caesarean delivery rate in each study group for nulliparous pregnancies, multiparous pregnancies, and all pregnancies. In general, *DS*_*all+np*_ recommended the most interventions for the three groups in nulliparous pregnancies, *DS*_*all*_ recommended the second most, and *DS*_*np*_ recommended the least interventions in the three groups. In contrast, *DS*_*all+np*_ recommended the least interventions in multiparous pregnancies, *DS*_*all*_ recommended the second most, and *DS*_*np*_ recommended the least interventions in the three groups. However, when combining nulliparous and multiparous pregnancies, the decisions made by *DS*_*all*_, *DS*_*all+np*_, and *DS*_*np*_ ∪ *DS*_*mp*_ on the healthy and acidosis group were not different. Finally, for all births, the combined *DS*_*np*_ ∪ *DS*_*mp*_ system recommended fewer interventions in the HIE group than the *DS*_*all*_ system (p = 0.015) and the *DS*_*all+np*_ system (p = 0.006). The recommendations of *DS*_*all*_ and *DS*_*all+np*_ on the HIE group were no different (p = 0.68).

**Figure 1:**
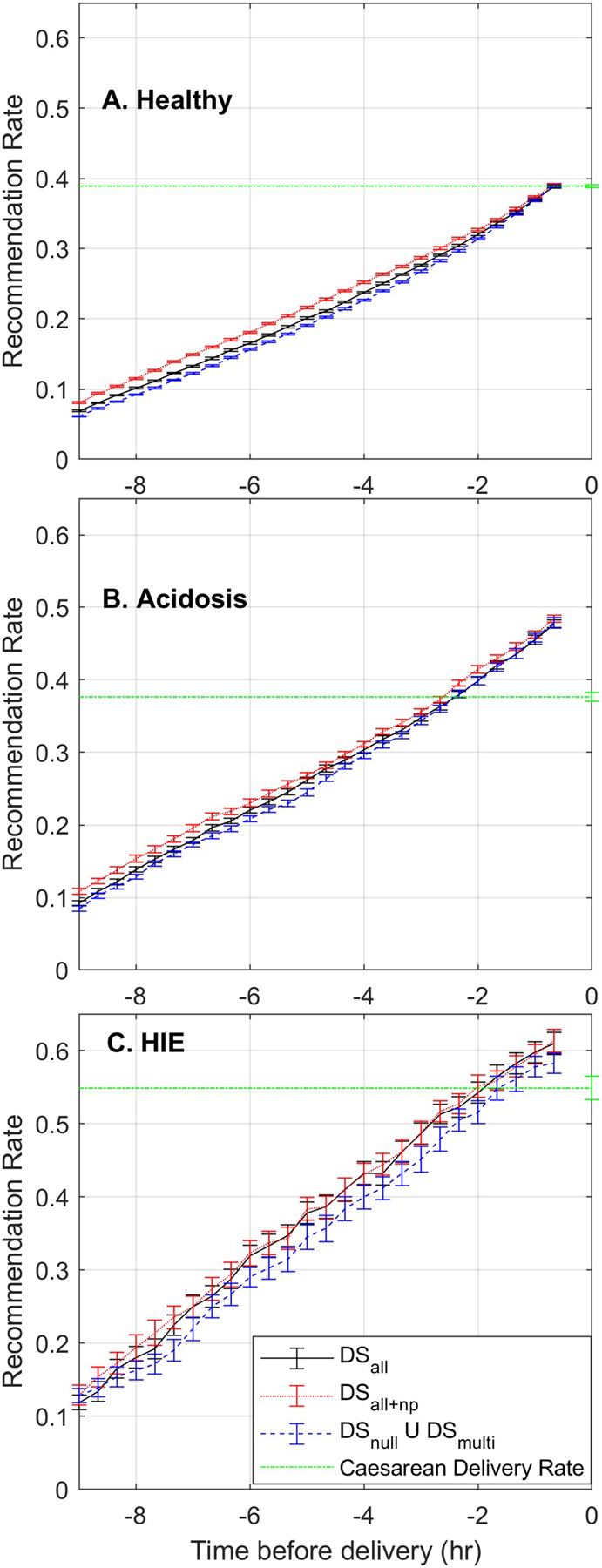
Recommendation rate of the *DS*_*all*_ system (black), *DS*_*all+np*_ (red), the combined recommendations *DS*_*np*_ ∪ *DS*_*mp*_ (blue) and the Caesarean delivery rate (green) as a function of time in the (A) healthy, (B) the acidosis, and (C) the HIE groups. The lines indicate the median of the recommendation rate, and the bars indicate the confidence interval of the median.

Fig. 1 shows the progression of the recommendations as a function of time before delivery made by the *DS*_*all*_ system in black, *DS*_*all+np*_ in red, and the *DS*_*np*_ ∪ *DS*_*mp*_ system in blue. The Caesarean delivery rate of each group is shown in green.

Fig. 1.A shows the progression of the recommendation rate for the healthy group; it was similar for the three systems, although up to 2 hr before delivery, the *DS*_*all+np*_ system tended to recommend more interventions. None of the classifiers ever recommended more interventions than the observed Caesarean delivery rate.

Fig 1.B shows the progression of the recommendation rates for the acidosis group; it was similar for the three systems, all three recommended more interventions than the observed Caesarean delivery rate starting 2 hr before delivery.

Finally, Fig. 1.C shows the recommendation rate for HIE cases. The *DS*_*np*_ ∪ *DS*_*mp*_ recommendation rate was always lower than that *DS*_*all*_ and *DS*_*all+np*_, although the confidence intervals of the medians overlapped most of the time. All systems recommend more interventions than the observed Caesarean delivery rate starting 80 min before delivery.

## V. Discussion

Our results showed that fetuses from nulliparous mothers were at an increased relative risk of developing acidosis and HIE. However, our results disproved the hypothesis that accounting for nulliparity, either via a two-path classifier or using it as an additional classification feature, could improve the detection of HIE.

The FHR results from the combined effect of several fluctuating influences mediated through mechanisms extrinsic and intrinsic to the fetal heart. Extrinsic mechanisms include the cardio-regulatory center in the fetal brain stem which receives input from the central nervous system, circulating catecholamines, and from peripheral receptors that are sensitive to oxygen level, pH (chemoreceptors) or stretch of a vessel caused by blood pressure changes. In response to these inputs, the cardioregulatory center can increase parasympathetic activity which reduces the FHR and increase sympathetic activity which causes peripheral vasoconstriction to support blood pressure and redistribution of blood flow to vital organs [9]. Intrinsic mechanics like myocardial hypoxia can also reduce the FHR.

There is no biological reason to expect that these anatomical and physiological pathways are different in a fetus according to its maternal history of childbirth. That is, given an equal degree of hypoxemia the fetal response should be similar in the fetus of a nulliparous woman compared to the fetus of a multiparous woman. Nulliparous women do have longer labors in both the first stage and in the second stage where contractions are the strongest and hence more likely to affect fetal oxygen levels. Thus, nulliparous labor can result in longer exposures to hypoxic conditions as well as other adverse conditions like chorioamnionitis [4, 10]. Indeed, nulliparity is associated with higher rates of HIE but the association is indirect. When considering FHR patterns which are a more direct reflection of actual fetal exposure to hypoxia and acidemia, we found no advantage to knowing maternal parity to develop a two-path classifier. In this context, our results are physiologically plausible. Furthermore, including the nulliparity variable in the one-path classifier did not improve the total detection of HIE either. The *DS*_*all+np*_ system interpreted that there was an increased risk of HIE in nulliparous women and recommended more interventions within that group when compared to *DS*_*all*_ and *DS*_*np*_ ∪ *DS*_*mp*_. Nevertheless, this was balanced by recommending less interventions in multiparous pregnancies and thus brought no advantage overall.

The effect of longer labors on the capacity of fetuses to withstand the stress of labor is still an issue that must be addressed in future experiments. Future work could address it by including the time from the onset of labor as a new feature. It is reasonable to expect that giving a time reference to a classifier could help it improve its interpretation of CTG patterns. Nulliparity is indirectly associated with the risk of HIE. Thus, it is also necessary to explore more direct risk factors and how to include them in the classification models.

## VI. Conclusion

Our study hypothesized that accounting for nulliparity could improve the detection of fetuses at increased risk of developing HIE. However, our results disproved this hypothesis: there was no advantage to separating the data compared to having a single classifier that included all pregnancies together or to include nulliparity as a classification feature. In the future, we will explore other clinical risk factors with a direct association to HIE and how to inform the classifier of the length of labor.

## Figures and Tables

**TABLE I.** DISTRIBUTION OF EACH STUDY GROUP WITH RESPECT TO THE LABEL OF NULLIPARITY.

	Nulliparous	Multiparous
*Healthy*	21,691 (57.8%)	15,855 (42.2%)
*Acidosis*	1,971 (64.5%)	1,085 (35.5%)
*HIE*	250 (66.8%)	124 (33.2%)

**TABLE II.** INTERVENTION RECOMMENDATIONS GENERATED BY THE DECISION SUPPORT SYSTEMS UP TO 40 MINUTES BEFORE DELIVERY. THE MEDIAN RATE OF RECOMMENDATIONS PER GROUP WITH THEIR CONFIDENCE INTERVALS. THE CAESAREAN DELIVERY RATE FOR EACH GROUP IS SHOWN FOR COMPARISON

	Nulliparous mothers		
	*Healthy*	*Acidosis*	*HIE*
***Caesarean delivery rate***	41.6% (41.4 – 41.8)%	34.5% (33.8 – 35.2)%	51.6% (49.2 – 53.9)%
***DS***_***all***_	46.8% (46.5 – 47.1)%	51.4% (50.9 – 52.0)%	64.3% (62.5 – 66.1)%
***DS***_***np***_	41.5% (41.3 – 41.8)%	48.3% (47.6 – 48.9)%	61.5% (59.7 – 63.3)%
***DS***_***all+np***_	56.0% (55.7 – 56.4)%	60.4% (59.6 – 61.1)%	72.9% (70.9 – 74.9)%
	Multiparous mothers		
	*Healthy*	*Acidosis*	*HIE*
***Caesarean delivery rate***	35.4% (35.2 – 35.6)%	43.0% (42.0 – 44.0)%	63.6% (60.9 – 66.4)%
***DS***_***all***_	28.2% (2S.0 – 28.4)%	40.8% (39.9 – 41.7)%	53.3% (50.5 – 56.2)%
***DS***_***mp***_	35.4% (35.2 – 35.7)%	47.5% (46.4 – 4S.6)%	55.1% (51.4 – 58.7)%
***DS***_***all+np***_	15.8% (15.4 – 16.3)%	26.4% (25.5 – 27.3)%	38.5% (35.7 – 41.2)%
	All births		
	*Healthy*	*Acidosis*	*HIE*
***Caesarean delivery rate***	38.9% (38.7 – 39.1)%	37.7% (37.1 – 38.3)%	54.9% (53.3 – 56.5)%
***DS***_***all***_	38.S% (38.6 – 39.0)%	47.7% (47.1 – 4S.3)%	61.0% (59.5 – 62.5)%
***DS***_***np***_ ∪ ***DS***_***mp***_	39.0% (38.8 – 39.1)%	47.9% (47.2 – 48.6)%	58.2% (56.9 – 59.6)%
***DS***_***all+np***_	39.1% (38.9 – 39.3)%	48.4% (47.9 – 48.9)%	61.3% (59.8 – 62.9)%

## Clinical Relevance—

Nulliparity is a well-known risk factor for developing hypoxic-ischemic encephalopathy. This study showed that there is no advantage in accounting solely for it in a classification system based on cardiotocography signals.
